# Comparison of optimized machine learning approach to the understanding of medial tibial stress syndrome in male military personnel

**DOI:** 10.1186/s13104-023-06404-0

**Published:** 2023-06-29

**Authors:** Vahid Sobhani, Alireza Asgari, Masoud Arabfard, Zeynab Ebrahimpour, Abolfazl Shakibaee

**Affiliations:** 1 Exercise Physiology Research Center, Lifestyle Institute, Baqiyatallah University of Medical Sciences, Tehran, Iran; 2 Chemical Injuries Research Center, Systems Biology and Poisoning Institute, Baqiyatallah University of Medical Sciences, Tehran, Iran; 3 Department of Physical Education and Sport Sciences, North Tehran Branch, Islamic Azad University, Tehran, Iran

**Keywords:** Bayesian optimization, Anthropometric predictors, Injury risk, Predictive methods

## Abstract

**Purpose:**

This study investigates the applicability of optimized machine learning (ML) approach for the prediction of Medial tibial stress syndrome (MTSS) using anatomic and anthropometric predictors.

**Method:**

To this end, 180 recruits were enrolled in a cross-sectional study of 30 MTSS (30.36 ± 4.80 years) and 150 normal (29.70 ± 3.81 years). Twenty-five predictors/features, including demographic, anatomic, and anthropometric variables, were selected as risk factors. Bayesian optimization method was used to evaluate the most applicable machine learning algorithm with tuned hyperparameters on the training data. Three experiments were performed to handle the imbalances in the data set. The validation criteria were accuracy, sensitivity, and specificity.

**Results:**

The highest performance (even 100%) was observed for the Ensemble and SVM classification models while using at least 6 and 10 most important predictors in undersampling and oversampling experiments, respectively. In the no-resampling experiment, the best performance (accuracy = 88.89%, sensitivity = 66.67%, specificity = 95.24%, and AUC = 0.8571) was achieved for the Naive Bayes classifier with the 12 most important features.

**Conclusion:**

The Naive Bayes, Ensemble, and SVM methods could be the primary choices to apply the machine learning approach in MTSS risk prediction. These predictive methods, alongside the eight common proposed predictors, might help to more accurately calculate the individual risk of developing MTSS at the point of care.

## Introduction

Musculoskeletal injury is a serious problem in the athletic world and military organizations. The prevalence of these injuries is very high and unacceptable [1, 2], about ~ 25% among military males [3] and 76% among all athletes [2]. This high prevalence places a necessary demand on injured subjects and, in general on societies to utilize healthcare facilities [4]. Medial tibial stress syndrome (MTSS) is one of the most common musculoskeletal injuries; the incidence rate of MTSS is from 4 to 35% in both military and sports medicine [5, 6]. Different studies mention a variety of risk factors such as navicular drop, body mass index (BMI), and age [7, 8]. However, the precise pathophysiology of this syndrome is not fully defined [8], and therefore prediction of this complex and multivariable syndrome is challenging. In addition to the burden of this syndrome, the recovery time is lengthy and extends from weeks to several months. Currently, MTSS is a highly recurring syndrome with no reliable treatment [9].

The machine learning (ML) approach includes strong analytical methods that could provide new insight into the interaction of variables. This approach has a significant potential to be used to manage injury risks in sports medicine [10]. Few studies have applied machine learning approaches in MTSS risk prediction or management [11–13]. Garnock and associates [13] used eight predictors and only utilized stepwise logistic regression for predicting the risk of injury. Other published studies have used ten independent risk factors and reported the ranked accuracies [9, 12]. However, they failed to fully address the importance of the predictors and optimized machine learning approaches for MTSS applications.

In the present study, the aim was to find an optimum ML approach capable of identifying influential predictors of MTSS in military recruits.

## Methods

**Fig. 1:**
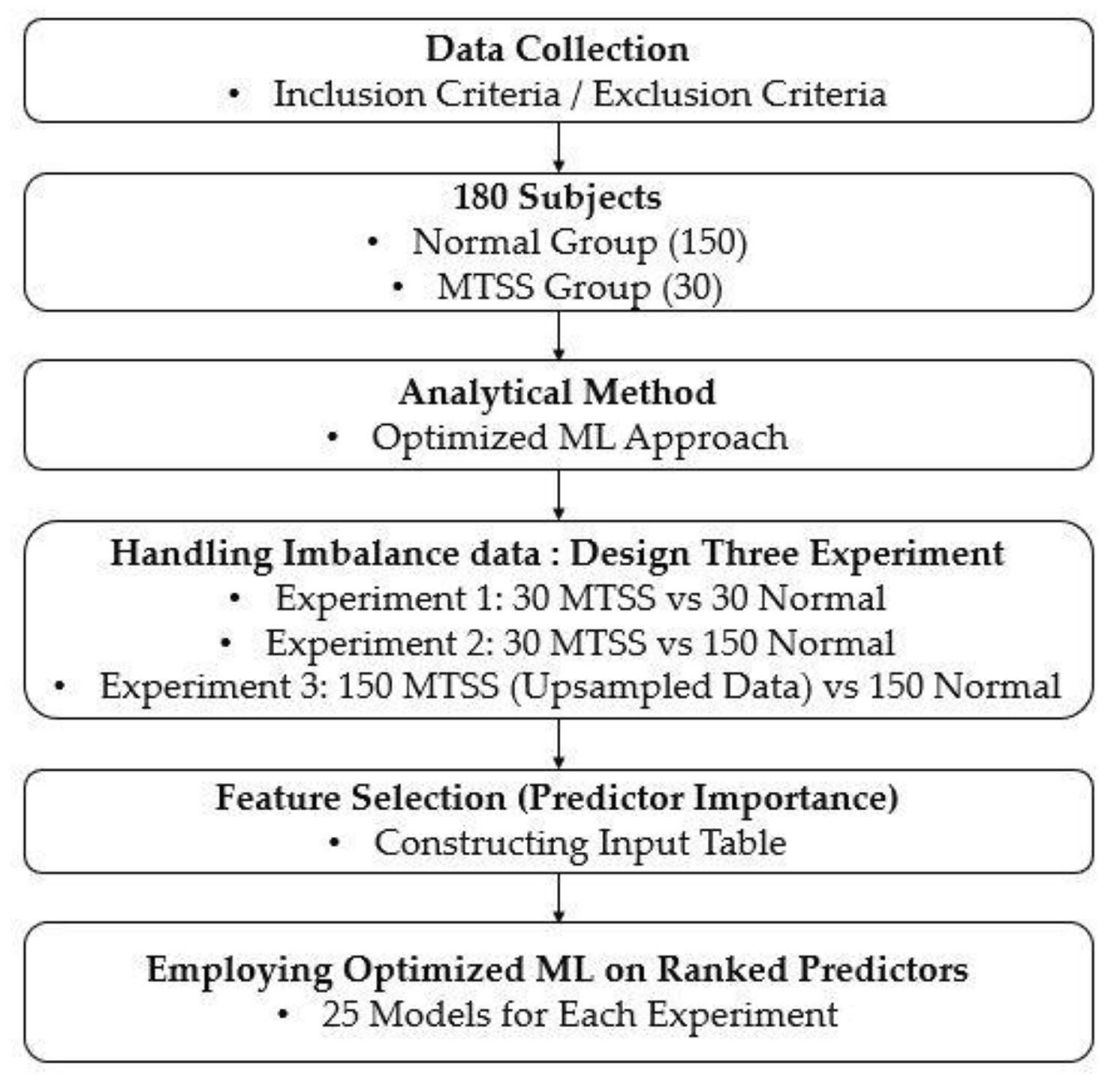
General block diagram of the method section

Two hundred male personnel during combat training in the infantry brigade enrolled in this cross-sectional study. The subjects signed a written consent form and were well informed about the project. After detailed consideration of exclusion criteria, 20 subjects have been excluded because of lower limb surgery, fracture history, and paresthesia symptoms. Therefore, the data of 180 recruits were included in the machine learning analysis. Out of 180 individual data sets, the no-injury (normal) group comprised of 150 and the MTSS group of 30. The study was performed after approval by the Ethics Committee of AJA University, Tehran, Iran. The Declaration of Helsinki was followed throughout the study. The MTSS was diagnosed according to the criteria put forth by Yates and White [14], including the appearance of pain following exercise lasting at least 2 h to several days. A general block diagram of the materials and methods section is presented in Fig. 1.

### Data collection

The predictors were measured twice in two alternate weeks. Each predictor value was recorded as the mean of two measurements. Predictors were of 3 categories: demographic, anatomical, and anthropometric. Age, weight, and height were acquired by four skilled specialists with an average of 4 years of experience as demographic data collectors. Seca stadiometer (Germany) was used to record heights barefoot (to the nearest 0.1 cm) weight was measured with a Seca scale (digital, Germany, seca 763) in barefoot and light clothing state (within 0.1 kg accuracy). Data are displayed in Table 1 in both complete and abbreviated form. Values are expressed as mean ± SD.

### Statistical analysis, machine learning approach

Machine learning algorithms transform the problem into an optimization problem to be solved normally [15]. The optimization problem comprises multiple hyperparameters that are set before the training process and defines how best the model fits the data. Random search has solved the expensive cost of exhaustive searching in grid search and proved more efficient in high dimensional space, even though it is not reliable for some complex models [16]. In addition, the problem of making the automatic tuning algorithm with high efficiency has not been fully solved in the machine learning approach.

Bayesian optimization solves the problem that a function has not a closed-form [17]. The algorithm comprises of two main steps (Eq. 2 and Eq. 3) introduced below where ${DATA}_{1:t-1}=\left\{{x}_{n}\right., {y}_{n}\}\genfrac{}{}{0pt}{}{t-1}{n=1}$ represents the training dataset with the *t-1* observation of unknown function.

Bayesian optimization workflow on the training dataset:

1. For *t = 1,2, …*.
2. Maximizing an acquisition function (a function) over f and finding the new point as
  1 $${x_t} = \arg \max a\left( {x|DAT{A_{1:t - 1}}} \right)$$

3. Updating the posterior distribution.

$${y_t} = f\left( {{x_t}} \right)$$

2 $$DAT{A_{i:t}} = \left\{ {DAT{A_{i:t - 1,}}} \right.\left. {\left( {{x_t},{y_t}} \right)} \right\}$$

4. End For.

Our problem, i.e., classifying the MTSS group vs. normal group using an optimized machine learning approach falls into this category, therefore, we used Statistics and Machine Learning Toolbox™ (MATLAB and Release 2020b, The MathWorks, Inc., Natick, Massachusetts, United States) for applying automatic machine learning methods with tuned hyperparameters. The optimization algorithm is already implemented in the machine learning toolbox of MATLAB software and can be used by employing the “fitcauto” function. This function automatically selects a subset of all possible learners, suitable for given predictor and response variables such as “ensemble”, “knn”, “svm”, “naïve bayes”, “tree”, etc.

The Bayesian optimization method in “fitcauto” internally includes a multi-TreeBagger model of the objective function. This method evaluates seven most applicable machine learning methods and automatically finds the best method with tuned hyperparameters on the training data. Once the optimization process is finished, the “fitcauto” returns the trained model on the entire train data set, which is expected to best classify new data [18].

### Imbalanced data sets in the machine learning approach

In our study, we had 30 MTSS subjects and 150 normal subjects, therefore, the uneven sample size imposes bias in machine learning methods training. To evaluate this drawback, we assumed three different experiments as undersampling, oversampling, and no resampling. First, 30 subjects were randomly selected from 150 subjects within the normal group to equate the two groups (undersampling). In the second experiment, we employed machine learning on the original imbalanced data (i.e., 30 MTSS subject’s data and 150 normal subject’s data) (no resampling). In the third case, the MTSS dataset group was randomly upsampled (i.e., generating 120 uniform distributed integer random values between 1 and 30) to 150 subjects, again equalizing the two groups but this time at 150 each (oversampling). Ultimately, the machine learning optimization approach was employed separately on constructed predictors and response data for each experiment.

### Table of predictors and response, feature importance

As mentioned in the data collection section, the study includes 25 predictors (Table 1) and the response column (0, normal group, 1, MTSS group). We used the filter type feature selection algorithm (e.g., feature ranking using F-tests) available in MATLAB software Statistics and Machine Learning Toolbox™. We ranked features’ importance as a preprocessing step and then trained the machine learning method by adding the next predictor considering the rank progressively. Twenty-five models were then generated for comparison in each experiment.

For each run, 85% of the dataset was used for training, and the remainder (15%) for the test step. The algorithm uses the k-fold (k = 5) cross-validation method to validate the training model. Output measures of our study to validate and estimate the effectiveness of each model were four well-known criteria, i.e., sensitivity, specificity, accuracy, and AUC.

## Results

As reported above, the prevalence of MTSS risk in our study was 16.16% (30 MTSS and 150 normal subjects). The table of the predictors (Table 1) contains 25 demographic, anatomical, and anthropometric variables.

**Table 1:** Twenty-five predictors were considered in the study for both MTSS (30 subjects) and normal (150 subjects) groups. Values are given as mean ± SD with significant differences between the two groups among the predictors. Significant predictors are marked with *

Predictor name (abbreviation)	Unit	Groups		P value
		**Normal** **(mean ± SD)**	**MTSS** **(mean ± SD)**	
Age	year	30.36 ± 4.80	29.70 ± 3.81	0.4791
Body Mass (BM)	kg	81.87 ± 14.21	79.77 ± 9.98	0.4415
Stretch Stature (SS)	cm	174.15 ± 7.94	173.96 ± 6.59	0.9024
Right Leg Length (RLL)	cm	87.48 ± 3.31	88.61 ± 4.70	0.1156
Left Leg Length (LLL)	cm	87.87 ± 3.55	88.60 ± 4.19	0.3202
Navicular Drop Test (NDT)	mm	6.19 ± 2.98	4.20 ± 3.13	*0.0011
Inter Condylar Interval (ICI)	cm	1.12 ± 1.51	1.31 ± 1.53	0.5310
Inter Malleolar Interval (IMI)	cm	2.00 ± 3.66	1.45 ± 3.01	0.4411
Q Angle (QA)	degree	14.40 ± 5.11	14.46 ± 5.51	0.9539
External Rotation (ER)	degree	45.36 ± 3.64	41.46 ± 3.73	*0.0001
Internal Rotation (IR)	degree	38.70 ± 5.02	37.03 ± 2.73	0.0788
Flexibility Right (FR)	degree	80.53 ± 11.35	81 ± 9.77	0.8327
Flexibility Left (FL)	degree	81.46 ± 10.10	78.33 ± 12.75	0.1407
Iliospinale Height (IH)	cm	51.15 ± 3.35	53.18 ± 2.96	*0.0024
Trochanteric Tibial Lateral Length (TTLL)	cm	43.02 ± 2.41	44.78 ± 1.56	*0.0002
Tibial Lateral Height (TLH)	cm	45.16 ± 2.54	46 ± 2.10	0.0913
Bi-illiocristalis (BI)	cm	30.64 ± 1.82	30.95 ± 1.41	0.3796
Femur Breadth (FB)	cm	10.08 ± 0.57	10.12 ± 0.26	0.7074
Calf Girth (CG)	cm	38.13 ± 3.25	38.55 ± 3.48	0.5239
Ankle Girth (AG)	cm	22.70 ± 1.42	22.71 ± 1.34	0.9717
Fat Percentage (FP)	percentage	20.16 ± 5.07	19.90 ± 5.24	0.7990
Fat Mass	kg	16.75 ± 6.24	15.81 ± 5.28	0.4416
Fat-Free Mass (FFM)	kg	65.20 ± 8.01	64.57 ± 6.45	0.6859
Fat Mass Right (FMR)	kg	2.37 ± 0.82	2.45 ± 0.91	0.6326
Fat-Free Mass Right (FFMR)	kg	11.47 ± 1.46	11.11 ± 1.27	0.2100

**Fig. 2:**
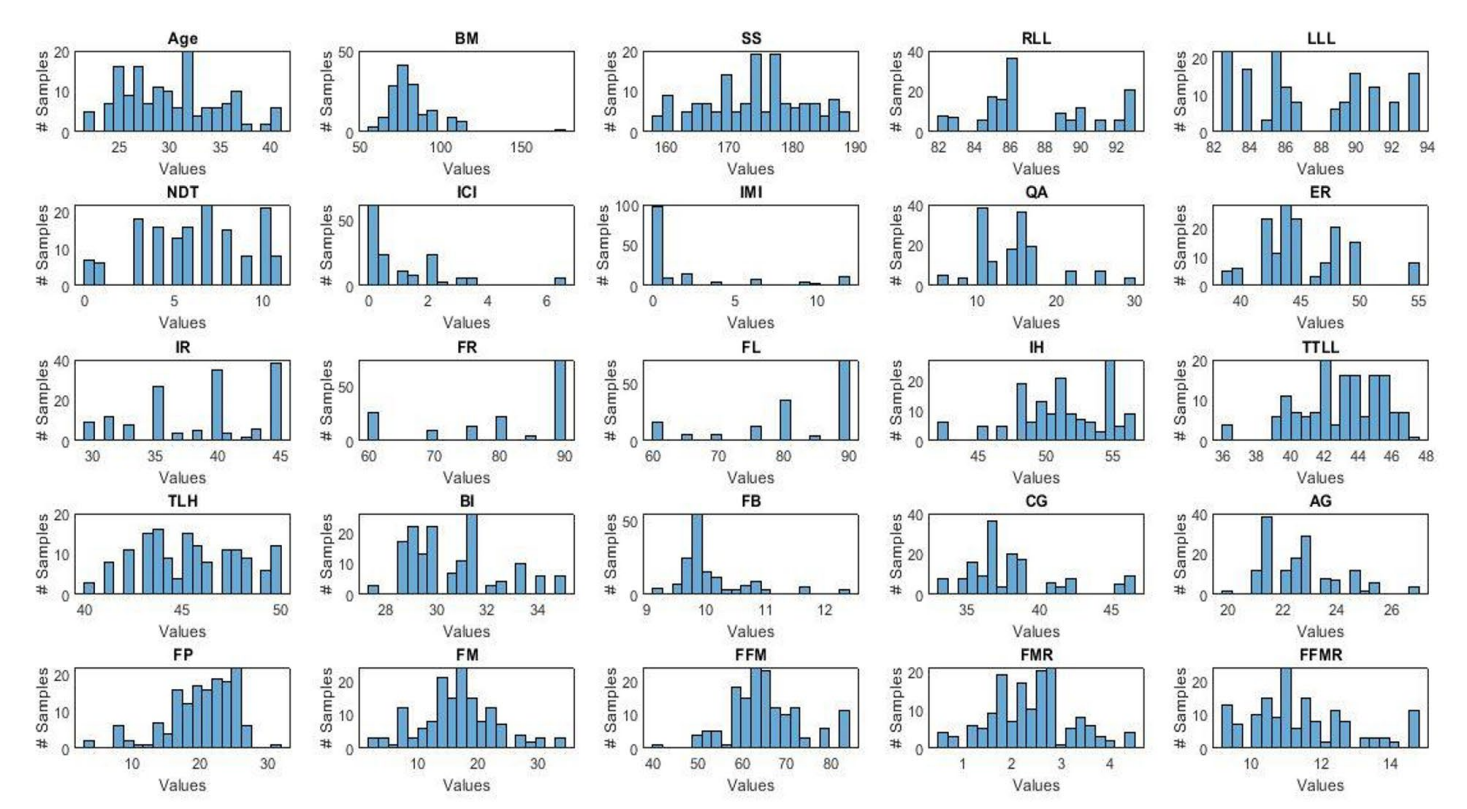
Histogram of the 25 predictors used for the normal group in experiments 2 and 3; Thirty subjects were randomly selected out of 150 normal subjects for no resampling experiment (Note: Blue color represents normal group)

**Fig. 3:**
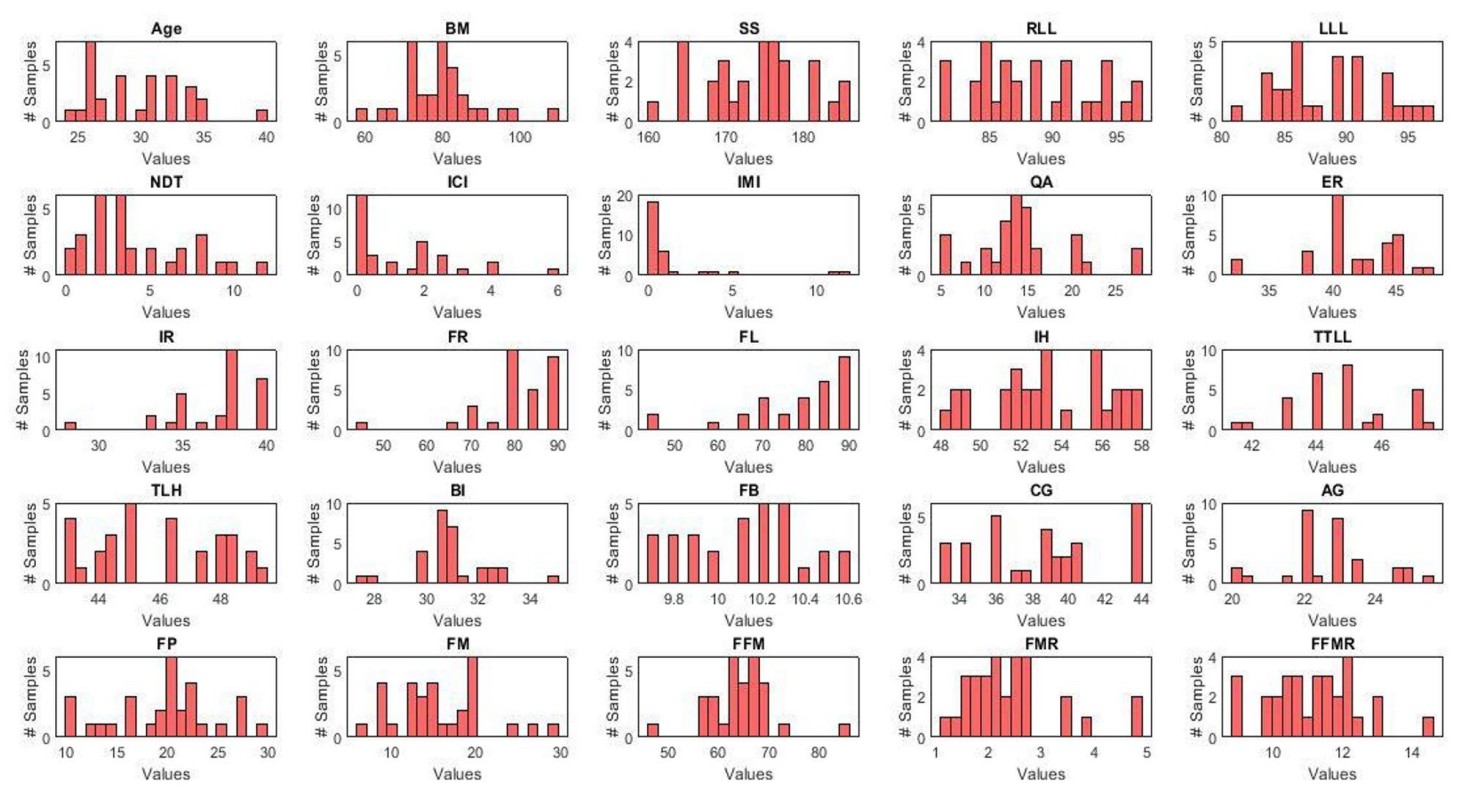
Histogram of the 25 predictors used for the MTSS group in undersampling, and no resampling experiments; In undersampling method the machine learning algorithm was employed on 30 normal and 30 MTSS subjects and in no resampling method the machine learning algorithm employed on 150 normal and 30 MTSS subjects. For oversampling method, these data were upsampled to achieve a 150 data sample size (Note: Red color represents MTSS group)

A Histogram of each predictor for both groups in three experiments (i.e., 30 vs. 30 as undersampling, 30 vs. 150 as no resampling, and 150 vs. 150 as oversampling) is shown in Figs. 2 and 3.

**Fig. 4:**
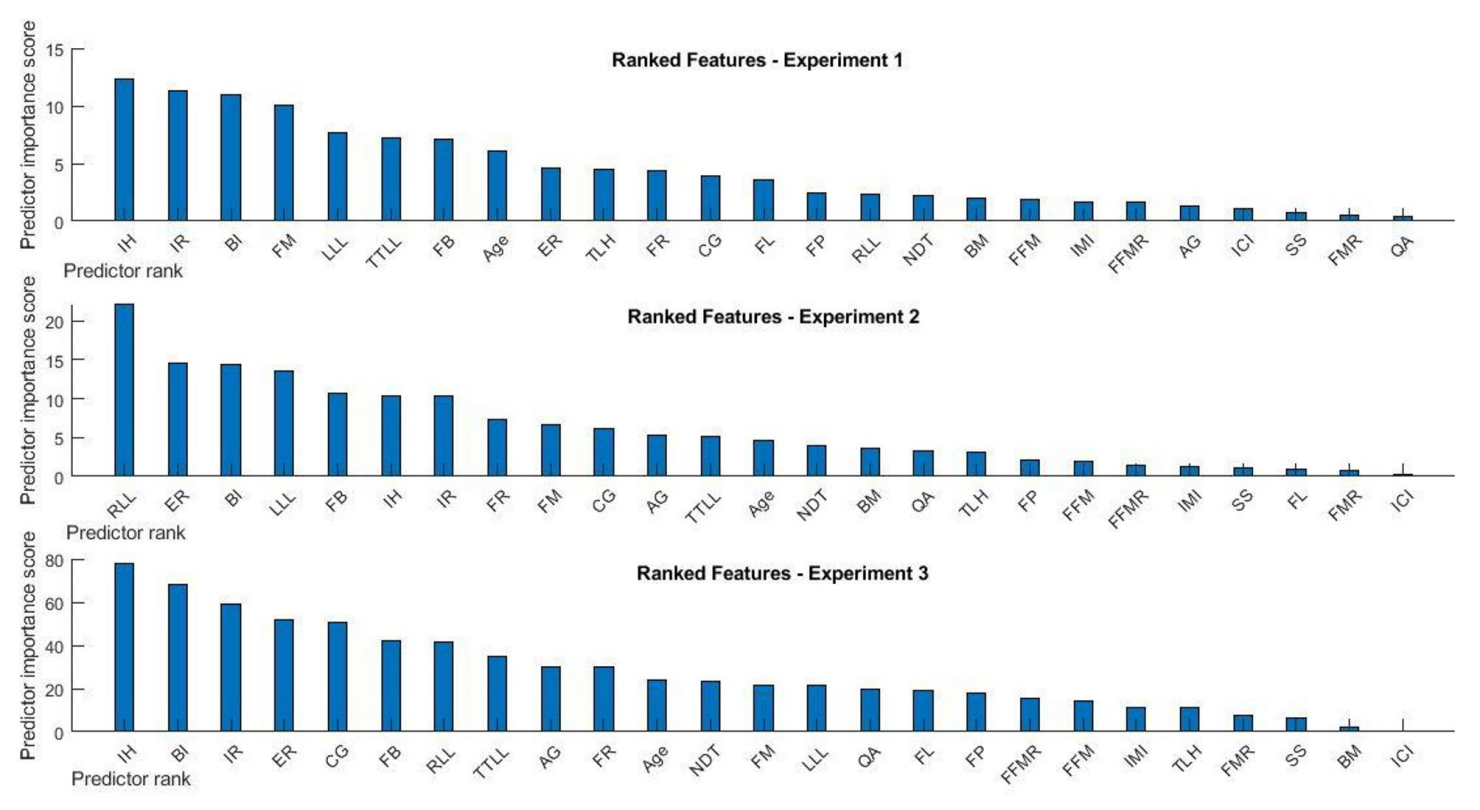
Predictor’s importance value and rank for undersampling experiment (Up), no resampling experiment (Middle) and oversampling experiment (Down); Examining the importance of each predictor individually using an F-test, and then rank features using the p-values of the F-test statistics calculated. The x-axis shows the predictor abbreviations/acronyms that have been sorted based on the rank and the y axis shows the importance value. The values in the y-axis are the negative logs of the p-values

We performed three different experiments; before each run, the table of predictors data was constructed, and the preprocessing step calculating the importance value of each predictor was done on the table of data. Relevant results of this step (i.e., ranked predictor names and importance values) for each experiment are shown in Fig. 4.

After the preprocessing of constructed predictor data set and sorting the predictors based on the calculated importance and rank, the optimized machine learning approach methods using the Bayesian optimization algorithm were implemented separately 25 times for each experiment. Results of the optimized model in each run were presented in Tables 2 and 3, and 4.

**Table 2:** The performance of employed optimized machine learning approach in undersampling experiment based on the predictor’s importance and the number of predictors

# Predictors	Accuracy (%)	Sensitivity (%)	Specificity (%)	AUC	Selected ML Model
1	44.44	33.33	66.67	0.722	KNN
2	77.78	66.67	100	1.000	Naive Bayes
3	66.67	66.67	66.67	0.889	Ensemble
4	77.78	83.33	66.67	0.944	Ensemble
5	77.78	83.33	66.67	0.944	Ensemble
6	100	100	100	1.000	Ensemble
7	100	100	100	1.000	Ensemble
8	100	100	100	1.000	Ensemble
9	100	100	100	1.000	Ensemble
10	100	100	100	1.000	Ensemble
11	100	100	100	1.000	Ensemble
12	100	100	100	1.000	Ensemble
13	100	100	100	1.000	Ensemble
14	100	100	100	1.000	Ensemble
15	100	100	100	1.000	Ensemble
16	100	100	100	1.000	Ensemble
17	100	100	100	1.000	Ensemble
18	100	100	100	1.000	Ensemble
19	100	100	100	1.000	Ensemble
20	100	100	100	1.000	Ensemble
21	100	100	100	1.000	Ensemble
22	100	100	100	1.000	Ensemble
23	100	100	100	1.000	Ensemble
24	100	100	100	1.000	Ensemble
25	100	100	100	1.000	Ensemble

**Table 3:** The performance of employed optimized machine learning approach in no resampling experiment based on the predictor’s importance and the number of predictors

# Predictors	Accuracy (%)	Sensitivity (%)	Specificity (%)	AUC	Selected ML Model
1	77.78	0	100	0.746	Naïve Bayes
2	85.19	50	95.24	0.766	Naïve Bayes
3	85.19	33.33	100	0.841	Naive Bayes
4	81.48	33.33	95.24	0.817	Naive Bayes
5	81.48	50	90.48	0.825	Naive Bayes
6	81.48	50	90.48	0.889	Naive Bayes
7	81.48	66.67	85.71	0.913	Naive Bayes
8	85.19	66.67	90.48	0.889	Ensemble
9	85.19	66.67	90.48	0.833	Naive Bayes
10	88.89	66.67	95.24	0.857	Naive Bayes
11	88.89	66.67	95.24	0.857	Naive Bayes
12	88.89	66.67	95.24	0.865	Naïve Bayes
13	85.19	50	95.24	0.802	Naïve Bayes
14	81.48	33.33	95.24	0.853	Naïve Bayes
15	85.19	50	95.24	0.786	Ensemble
16	85.19	50	95.24	0.802	Ensemble
17	85.19	50	95.24	0.849	Ensemble
18	85.19	50	95.24	0.825	Ensemble
19	81.48	33.33	95.24	0.825	Ensemble
20	85.19	33.33	100	0.929	Ensemble
21	81.48	33.33	95.24	0.786	Ensemble
22	85.19	50	95.24	0.873	Ensemble
23	85.19	50	95.24	0.889	Ensemble
24	81.48	33.33	95.24	0.825	Ensemble
25	81.48	33.33	95.24	0.929	Ensemble

**Table 4:** The performance of employed optimized machine learning approach in oversampling experiment based on the predictor’s importance and the number of predictors

# Predictors	Accuracy (%)	Sensitivity (%)	Specificity (%)	AUC	Selected ML Model
1	71.11	77.78	66.67	0.728	Tree
2	91.11	100	85.19	0.907	SVM
3	100	100	100	1.000	SVM
4	100	100	100	1.000	SVM
5	100	100	100	1.000	SVM
6	100	100	100	1.000	SVM
7	100	100	100	1.000	SVM
8	95.56	88.89	100	1.000	SVM
9	95.56	88.89	100	1.000	SVM
10	100	100	100	1.000	SVM
11	100	100	100	1.000	SVM
12	100	100	100	1.000	SVM
13	100	100	100	1.000	SVM
14	100	100	100	1.000	SVM
15	100	100	100	1.000	Naive Bayes
16	100	100	100	1.000	SVM
17	100	100	100	1.000	SVM
18	100	100	100	1.000	SVM
19	100	100	100	1.000	SVM
20	100	100	100	1.000	SVM
21	100	100	100	1.000	SVM
22	100	100	100	1.000	SVM
23	100	100	100	1.000	SVM
24	100	100	100	1.000	SVM
25	100	100	100	1.000	SVM

The results show the high-performance rate of using a machine learning approach for classifying the MTSS subjects from the normal group. In the undersampling experiment, the accuracy rate, sensitivity, and specificity of 100% were obtained for the Ensemble classification model when using at least the six most important predictors (features).

For no resampling experiment, the best validation results (accuracy = 88.89%, sensitivity = 66.67%, specificity = 95.24%, and AUC = 0.8571) were achieved for the Naive Bayes classifier while the predictor data table consisting of the ten and/or 11 or 12 most important features (see shaded rows in Table 3).

In the oversampling experiment, the best validation parameters with the highest performance (100%) were observed while using only the three most important predictors (see the third shaded row in Table 4). Then, we witnessed a decay in the performance when adding the eighth and ninth predictors to the predictor’s data table (see 8 and 9 shaded rows in Table 4). Further along, the performance was increased again while at least ten predictors were used to construct the predictor’s data table (see the 10th shaded row in Table 4). The support vector machine classifier has the best performance in oversampling experiment.

## Discussion

The goal of the study was to evaluate the optimized machine learning approach to predict MTSS incidence out of many demographics, anatomic, and anthropometric measured variables. As the results show, efficient performance, even 100%, was achieved for predicting the new cases as normal or MTSS considering underlying patterns of predictors interaction/combination with the optimized machine learning approach. The problem of how to make or use an automatic algorithm with tuned hyperparameters is a challenge [19]. Therefore, in this investigation, the Bayesian optimization approach was used to find the optimum machine learning algorithms among eight important machine learning methods for predicting the risks leading to MTSS. Despite the applicability and strong qualities of machine learning in predicting the potential risks, handling imbalances in a dataset is crucial, which has not been examined in relevant studies [9, 12]. To handle this drawback, we implemented three separate experiments and in the oversampling experiment the MTSS dataset was randomly upsampled to 150 subjects.

Furthermore, the study results help to propose selecting the main predictors contributing most to the emergence of MTSS using the feature selection method and running the machine learning approach by progressively adding a remained new predictor 25 times. In the undersampling experiment, the highest accuracy (100%) was achieved using at least six most important predictors (i.e., IH, IR, BI, FM, LLL, and TTLL) (Table 2; Fig. 4). Results of no resampling experiment emphasize the effect of the combination of 10 and/or 11 and 12 most important predictors with the highest obtained performance (i.e., RLL, ER, BI, LLL, FB, IH, IR, FR, FM, CG, AG, and TTLL) (Table 3; Fig. 4). The best-selected model (e.g., Naive Bayes) for our optimization machine learning approach in no resampling experiment is in line with the best accuracy rate of two recent studies [9, 12]. Oversampling experiment results demonstrate that in the best case using the three most important predictors could provide the best performance, and also using the combination of 10 predictors shows the highest accuracy, sensitivity, and specificity (i.e., IH, BI, IR, ER, CG, FB, RLL, TTLL, AG, FR) (Table 4; Fig. 4). The best-optimized machine learning algorithms that obtained for the undersampling experiment and oversampling experiment are the ensemble classification model and SVM classifier, respectively. While the results confirmed the hypothesis that both anatomic and anthropometric predictors have an essential contribution to estimating the risk of MTTS incidence, the collection of all 25 variables is both expensive and time-consuming, especially when the target population is large.

These features are not in contradiction to the relevant studies. Biomechanical dysfunction, including IH was reported as a suspected variable in the development of MTSS [20]. Internal hip rotation has a significant relationship with MTSS, and external hip rotation is reported as a primary risk factor for MTSS [21, 22]. Our results also confirm the importance of these two features, and we emphasize acquiring IR and ER in subsequent studies. Further, several studies mentioned FM as the main risk factor [23]. Biomechanical studies introduced the lower limb length and other anthropometric predictors as possible risk factors in MTSS, but the exact relation or significance is not well defined [24]. The results of this study propose the anthropometric parameters (e.g., BI, TTL, CG, and FB) as risk factors alongside other predictors.

There are limitations in our study; the study only considered the male personnel, as the combat brigades are all-men infantry units. The prevalence and predictor’s importance or weight might turn out to be different in female populates [25, 26]. Since the recruits were affiliated with a single military unit, results and conclusions hereby drawn are not to be generalized to other non-military populations [13, 27]. Future studies are needed to examine the replication power of this method on otherwise matched populations.

## Conclusions

Employing optimized machine learning approach method offers a more accurate risk prediction model for MTSS syndrome. The Naive Bayes, Ensemble, and SVM could be the first choice for future studies. IH, IR, BI, FM, TTLL, CG, FB, and ER variables are important predictors to concentrate upon for future studies. These predictors and predictive methods might help military medicine officers and sports medicine clinicians to more accurately calculate the risk of MTSS at individual level at the point of care.

## Data Availability

The datasets generated and/or analyzed during the current study are not publicly available due to privacy policy but are available from the corresponding author upon reasonable request.
